# The Hospital Nursing Supplement

**Published:** 1892-09-17

**Authors:** 


### "The Hospital" Nursing Mirror.

Being the Extra Nursing Supplement of "The Hospital" Newspaper.

Contributions for this Supplement should be addressed to the Editor, The Hospital, 140, Strand, London, W.C., and should have the word "Nursing" plainly written in left-hand top corner of the envelope.

### En Passant.

A TRAINED NURSE FOR SIDCUP.—The medical men of Sidcup have all expressed approval of Mr. Shapley's scheme for securing a thoroughly-trained district nurse for the neighbourhood. The suggestions made in a letter in the *Sidcup Times* are eminently practical, and we hope the plan will be speedily and successfully carried out.

THE VICTORIA HOSPITAL FOR CHILDREN, CHELSEA, is appealing to its friends through the medium of a little poem, by the Secretary. It is illustrated with some very pretty pictures, which will certainly attract attention to the needs of "The Coster's Little 'Un," and we hope that they will also aid the funds of this deserving hospital.

PRINCE ALFRED HOSPITAL, SYDNEY.—The annual report tells us that the new Nurses' Home is approaching completion, and we can well believe that it will be worthy of the beautiful hospital with which it is connected. Funds for furnishing the house are still needed, but we cannot believe that the liberal Australians will allow this state of things to continue. The empty rooms will be soon transformed into comfortable home-like apartments, and the nurses who tenant them will be appreciative of the thought and trouble cheerfully devoted to considerations for their well-being.

WITHERED WINDOW PLANTS.—When "everybody" is out of town, the unfashionable working "nobodies" manage to go on very contentedly, doing their daily tasks without missing the absentees much, if at all. But before "everybody" goes away next year, it would be very nice if orders were given to transfer the window plants to some of the hospitals and infirmaries, where they would not only be welcomed, but also carefully tended. It is really a great trial to flower-lovers to see the window boxes, outside the shuttered panes of many-storied West-End houses, grow more shabby day by day, until dead leaves and withered stalks alone remain, in ghastly mockery of the gay blossoms of the past season. Sympathy for the sick or poor "nobodies" would cancel such waste of beauty.

A PRETTY PICTURE.—Walking down one of the quiet streets in Royal Windsor on a sunny afternoon, thinking, maybe, of the fine castle just visited, and the glorious prospect viewed from the historic Terrace, our steps were arrested, and our reflections diverted into another channel by an altogether different sight. A little child, undoubtedly "a hospital case," for its head was bandaged, looked out at us from a bay-window where a nurse in uniform also appeared, holding another small patient tenderly in her arms. There was an assortment of flowers, too, but the little human plants were evidently in no danger of being eclipsed by the window-boxes. Of course we drew near, and discovered that the unpretentious brick building was dignified by the title of "Royal Infirmary," and we were soon pleasantly welcomed and introduced to the orderly interior of this bright little hospital. The children's ward was very attractive with polished floor, and great bow-window forming one end of the room. The brass cots were the best we had ever seen, as regards their construction, but we were sorry to find that the names attached to them only referred to the donors of the bedsteads, and that they are all unendowed. The other wards were in equally spotless condition, and the whole place seemed to afford evidence of good work efficiently achieved.

SHORT ITEMS.—The Empress of Germany has an English trained nurse in attendance on her.—Miss Priestman and some other ladies in Hull are going to organise a nursing institution for the sick poor.—Dr. Elizabeth Blackwell has written a pamphlet on "Why Hygienic Congresses Fail." She seems to think the reason, at any rate, lately, has been the undue prominence given to bacteriology, to the exclusion of practical hygiene.—It is proposed to hold a drawing-room sale of work in December for the benefit of the Trained Nurses' Club and Library, contributions will be gratefully received.

LADY LECTURERS.—The cottagers in far-away country villages are tasting the fruits of modern civilization without always appreciating their highly-refined flavour. We heard of a small and attentive audience being very much puzzled by a recommendation to use Loofah for their children's baths. Its advantages were detailed, but its appearance was not described. "What ever can her mean by Loo-fer; do ye think it's some newfangled soap?" said one of the mothers on her way home. "Well, I reckon it's all she can get in London, poor girl. She aint much of a colour to look at, Betsy. I've half a mind to tell her she'd be a sight prettier if she went in for yellow soap and plenty of water."

THE FORESTERS AND SALISBURY INFIRMARY.—Hospital Saturday was chiefly noticeable in the Cathedral City from the presence of the collecting boxes, which were placed in the hotels and in all places of public resort, and appeared to be receiving substantial support from the passers-by during the day. Hospital Sunday was marked by a great demonstration of Foresters, who marched through the town, attended by the 1st Wilts Rifles, the drum and fife, and the Salisbury town bands. A service at the Congregational Chapel was largely attended by the thrifty working-class, which has benefited so extensively by the infirmary. It is pleasant indeed to find them so willing to bear witness to the good done within those hospital wards, and so anxious to show their gratitude by this cordial public demonstration and collection. We hope to hear of a substantial addition being thus made to the year's receipts.

NURSES' RECREATION.—Most people are, and all ought to be, ready to own that "healthful play" forms quite as essential a part of life as the work which is the lot of most of our readers. But if "play" is to prove literally "recreation," it must be suitable to the individual players. Those who lead sedentary lives need active sports, but to active workers, such as nurses especially, the amusements required are restful ones. Drives, or sailing, if there is water within reach, and all kinds of entertainments which can be enjoyed without bodily fatigue, these are suitable for "recreation." Young probationers and sisters of small wards enjoy an occasional game of tennis, but no one who is tired by work will care to fatigue herself additionally by play. She wants rest—mental change and bodily ease—and the wise woman learns how to gain both. Dances for nurses are especially out of place, simply because no one can possibly be fresh and fit for onerous duties in the early morning hours when she has fagged herself out over night. Let dances be the suitable and agreeable ingredients of the annual holiday, when it is good to indulge in every variety of healthy pleasure; but they don't mix well with our daily life amongst the sick and sorrowful. Patients have a right to the services of fresh, bright, and willing nurses, and those who do not choose their recreation aright soon cease to merit these descriptive terms.

### How to Become a Medical Woman.

*(Continued from page clxxi.)*

The first thing the would-be medical student has to do, after deciding at which qualification she means to aim, is to pass the necessary examination in arts recognized by the Medical Council, the successful passing of which will enable her to be registered. If she has decided to try for a university degree, the matriculation examination of the university selected must be passed. London University holds matriculations twice yearly, in June and January in London, and also in a certain number of provincial towns, of which a list can be obtained from the Registrar, University of London, Burlington Gardens, W. The Irish University holds its matriculation in October only, in Dublin, Cork, and Belfast, and the student can obtain particulars of the range of subjects, and of the prescribed authors in Latin, &c., from the Secretary, Earlsfoot Terrace, Dublin.

For those students who wish for the qualification granted by the Apothecaries Society of London, or by the Scotch or Irish colleges, the examinations in arts of the Apothecaries, or of the College of Preceptors, is accepted, so long as a first or second class is obtained, and the proper subjects are selected. These are English grammar, Latin, arithmetic, Euclid, books I., II., and III., algebra up to quadratic equations, mechanics, and one other language, either French, German, or Greek, at the option of the student. It is very important to remember, that by a recent regulation of the Medical Council, all these prescribed subjects must be passed at once, and no credit is given, as formerly, for any single subject passed. Both the apothecaries and the preceptors hold an examination every quarter, early in March, June, September, and December; particulars can be obtained from the Secretary, at Apothecaries' Hall, Blackfriars, E.C., and College of Preceptors, Bloomsbury Square, W.C., respectively. The examinations held by the Preceptors in March and September are stated to be especially for medical students, whilst those in June and December are for schools; but so long as the medical student chooses the right subjects and gets a first or second-class, these last will serve the purpose. As soon as the necessary arts examination is passed, the student should without delay apply to the Secretary of the General Medical Council, Oxford Street, W., for the necessary forms to fill in for registration as a medical student, so as to get her name entered on the register. After this, the student enters one of the women's medical schools and begins her "professional studies," and prepares for the first examination. We have given in tabular form for easy comparison the various subjects required by London University for its "Intermediate Examination in Medicine"; by the Irish University's "Second Examination in Medicine"; and by the Scotch and Irish colleges and apothecaries for their "First Professional," as at present constituted; but it must be borne in mind that both the colleges and the apothecaries have extended their curriculum from four to five years, and have made biology compulsory. It is to be hoped the new regulations will be issued before the opening of the session in October. The alterations will not materially alter the course, but tend to spread it over more time, and divide up the subjects, more after the manner of the universities, which already have the five years' curriculum.

| London University. "Intermediate." | Irish University. 2nd Examination in Medicine. | Irish Colleges. 1st Professional. | Scotch Colleges. 1st Examination. | Apothecaries. Part I. of "Primary." |
|---|---|---|---|---|
| Anatomy.<br>Physiology.<br>Histology.<br>Materia Medica.<br>Pharmaceutical Chemistry.<br>Organic Chemistry. | Anatomy.<br>Physiology.<br>Materia Medica.<br>Pharmacology.<br>Practical Chemistry. | Physics.<br>Chemistry.<br>Practical Chemistry.<br>Practical Anatomy and Osteology.<br>Practical Pharmacy.<br>Biology? | Chemistry.<br>Practical Chemistry.<br>Histology.<br>Practical Anatomy and Osteology.<br>Biology? | Chemistry, with Chemical Physics (Heat, Light, and Electricity).<br>Practical Chemistry.<br>Materia Medica and Botany.<br>Pharmacy.<br>Biology? |

Before entering for the above examinations, the London University requires two years, and each of the other examining boards one year's study of the prescribed subjects, *i.e.*, one medical year, which comprises a winter and summer session. The London University holds no other examination until the M.B., two years later, but all the others have yet another before the final, which we have also tabulated.

| Irish University. 3rd Examination in Medicine. | Irish Colleges. 2nd "Professional." | Scotch Colleges. 2nd Examination. | Apothecaries. Part II. of "Primary." |
|---|---|---|---|
| Anatomy.<br>Physiology.<br>Histology. | Anatomy.<br>Physiology.<br>Histology.<br>Materia Medica.<br>Medicine.<br>Elementary, Medical, and Surgical Hospital Practice. | Anatomy.<br>Physiology.<br>Materia Medica.<br>Pharmacy. | Anatomy.<br>Physiology.<br>Histology. |

This examination must in all cases be preceded by one year's study. After this comes the "final" in surgery, medicine, pathology, midwifery, &c., and so completes the full course for qualification.

We have now to consider the various medical schools where women can obtain the necessary teaching, the courses of lectures provided by these, and the fees charged. In London there is the London School of Medicine for Women, Handel Street, Brunswick Square, W.C. In Edinburgh there has, unfortunately, been a split in the medical world, so far as women are concerned, and as a result the two schools are neither of them very adequately supported by funds which were only intended to endow one school. These are the Medical College for Women, 30, Chambers Street, Edinburgh, and the Edinburgh School of Medicine for Women, Surgeon Square. In Glasgow there is St. Margaret's College. We will give the fees for the school course and hospital practice at each of the above in tabular form; also a list of extra fees for special subjects, and as these fees vary in some cases according to the number of the students, we have quoted the minimum in each case. In addition, books, instruments, a set of osteology, and a microscope, together with a certain quantity of chemical apparatus, and materials for histology, such as slides, stains, &c., must be added, and are said to vary from £10 to £30. The student must not forget that she will very probably need private "coaching," especially if she aims at a university degree, and in most cases it will be required in chemistry if this subject has not been studied previously to entering the school.

Fees of Various Schools.

| London School. | Edinburgh College. | Edinburgh School. | Glasgow College. |
|---|---|---|---|
| School and Hospital, for whole course, £105<br>Extras.<br>Operative Surgery, £3 3s.—£5 5s.<br>Practical Pharmacy, 3 months, £1 1s.<br>Practical Midwifery, £5 5s.—£16 16s.<br>Operative Midwifery, £3 3s.<br>Vaccination, £1 1s.<br>Instruction in Anaesthetics, £1 1s.<br>Total extras £14 14s.<br>Total fees, £119 11s. | School and Hospital, for whole course, £80 | School and Hospital, for whole course. £86<br>Extras, £12—£14.<br>The prospectus states, however, that the total fees may be estimated at £120.<br>Total fees, £120. | School and Hospital, for whole course, £80<br>Extras, £10—£20.<br>Total fees, £90. |

Thus, by simply adding together the total fees of the school and examining board selected, and the probable cost of books, &c., together with possible fees for "coaching," each student can estimate approximately the cost of a qualification as at present arranged. The extra cost of a five years' curriculum can easily be added to the above.

There seems every prospect that very shortly women will be able, under certain conditions, to obtain a medical degree at some of the Scotch Universities. It would be premature at this time to give these conditions, but would be worth while for all students to take this into consideration in making arrangements.

*(To be continued.)*

### Ladies' Conference at the Sanitary Congress.

THE crowded and enthusiastic audience which filled the room assigned to the Ladies' Conference in the Town Hall, Portsmouth, last Tuesday, is a fresh proof of the importance which is beginning to attach itself to the subject of domestic hygiene. The Congress of the Sanitary Institute must feel repaid for its courteous encouragement to ladies to co-operate when they find their invitation so cordially accepted. Much of the success of the Conference was due to Mrs. Day's (the President) able organisation. Not only were the papers read of unusual value, but the discussions on each subject were also conducted with admirable spirit. The Town Hall at Portsmouth is a very fine modern building, and the room reserved for the ladies' section held about two hundred people, but the ventilation was certainly not adequate for an audience of that size. The air was heavy, and the temperature deplorably high before the expiration of the first hour, and it served as a practical example of the need for fresh air, which some of the speakers eloquently advocated.

Sir Charles Cameron inaugurated the proceedings in a kindly and amusing speech, and considered this new departure a valuable auxiliary to the sanitary department. He spoke of the practical work done in other large towns by ladies, and especially mentioned the excellent results obtained by their aid in Dublin.

Mrs. Day referred to the teaching and example of Miss Nightingale, and spoke of Charles Kingsley's celebrated address to the Ladies' Sanitary Association thirty years ago, when the excessive infant mortality was stigmatised by him as "massacre." Miss Ethel Lamport read an excellent paper on "Food, with Special Relation to the Sick," which was listened to with marked attention. In the discussion which followed, Miss Edith Barnett made some suggestions with regard to the dieting of children, and Sir Charles Cameron responded with much humour, expressing very decided views against little folks being deprived of all their favourite articles of food, more especially sugar. He decried the old-fashioned views respecting the harm done by sweets, and also spoke warmly of the dietetic modern fads, the perusal of literature bearing on the subject giving him often "excruciating agonies." Whatever differences of opinion might exist on the question, of white or whole-meal bread, everyone cordially applauded Sir Charles' assertion that "Nature is a far better physiologist than man," and, therefore, "the instinct of man generally finds out, in the long run, what is best for him." Dr. Williams Freeman read a paper on "Chief Hygienic Causes of Infant Mortality," which was extremely interesting and instructive. He has evidently bestowed considerable attention on the subject, and holds very strong views on what to do and what to leave undone in the bringing up of young children, and he spoke with admirable energy of the healthy modern mothers who can, but will not, nurse their own babies. Clothing and other subjects were also briefly referred to with much practical common sense by Dr. Williams Freeman. Miss Pilkington joined in the discussion with great spirit and told amusing anecdotes of her personal experiences. Miss Charlotte Smith spoke on the important subject of "Physical Exercises," and Miss Barnett had much to say about fresh air in houses, and advocated "draughts," which Dr. Ward Cousens later on aptly termed "the acute form of ventilation." Dr. Schofield spoke on "The Necessity for Home Education in Hygiene," and the proceedings concluded with a cordial vote of thanks to Mrs. Ernest Day, proposed by Sir Thomas Crawford, and seconded by Miss Barnett.

### Our Australian Letter.

ALL kinds of things attract our attention, pleasantly and unpleasantly, too, and the term Baby Farming belongs to the second class. It is incongruous; it therefore jars on the nerves of every humane man and woman. Many things may be "farmed" to their own and their possessors' advantage, but babies are not things, and they need tenderer treatment, and some day, perhaps, they will be better protected. At present the most we seem able to do is, occasionally, to secure legal punishment for the woman who has illegally allowed a slow and sure death to overtake the helpless creatures which fate has put within her power. It is seldom that any steps are taken to save the infants from lingering starvation; it seems "nobody's" business to interfere until after the end desired by parent and "farmer" is attained!

The last case which has roused the indignation of newspaper readers was reported in the *Argus* of June 18th, and we hear that "the child in question was literally starved to death in three weeks, and the medical evidence was that it died from insufficient and improper feeding." His Honour, Mr. Justice Hood, in summing up the case, after a spirited speech from the Crown Prosecutor, explained that Mrs. Elizabeth Perry was an extremely poor woman; that she had three other unfortunate infants confided to her; that she took a lodger, and her household consisted in all of seven persons, inhabiting a wretched two-roomed hovel. She was said to "adopt" the babies, which meant, in common English, that she received sums, varying from £10 to £3, paid by their natural guardians, for her to take the children entirely off their hands. A charge of manslaughter is a serious one, whatever the verdict of the jury may be; but so long as Australian or English mothers are willing to pay, just so long will wretched women be found, tempted by their poverty, to face the risks of criminal trials as a sequence to their speculations in "Baby Farming."

At the Women's Hospital at North Carlton a good report of cases and of general health was given at the monthly meeting (June). A discussion was held on the finance question, it being proposed to reduce the salaries of officers, nurses, and servants. This appears to be such a serious as well as unusual step that we shall be glad to hear more on the matter. It is impossible for a just opinion to be formed by the public unless some official statement of the salaries granted at present be brought forward for comparison with the proposed rates of payment.

The arrangements for quarantine, fumigations, &c., during the visitation of small-pox appear to have been satisfactory and sufficient, and no doubt it is owing to the precautions taken that no spread of the epidemic in the colonies has occured.

The first annual meeting of the Melbourne Medical Association was held on June 30th, and the President spoke well on the subject of vaccination, at present one of burning interest in the colonies. He thought it would be well to follow the English plan of granting a bonus to every public vaccinator in proportion to his percentage of efficient vaccinations.

At a meeting of the Australian Health Society in June the Ladies Committee reported in the course of the proceedings that a series of health lessons, conducted by Dr. Clara Stone at South Melbourne, had been most successful and popular. The question of domestic hygiene appears to be attracting intelligent interest all over the world, and we may hope to find women becoming universally "healthy," as well as "wealthy and wise," in this progressive nineteenth century.

### Our Indian Letter.

The annual statement of accounts issued by Lady Roberts in connection with her Fund shows how hopeless it would be for any private enterprise of a similar kind in India to become a finanical success.

Statement of Receipts and Expenditure.

| *Receipts.* | Rs. | a. | p. |
|---|---|---|---|
| Balance in hand on 1st July, 1891 ... ... | 5,759 | 10 | 0 |
| Payments by patients in officers' hospitals, by Government nurses on leave in the homes, and by patients nursed in their own homes... | 5,154 | 3 | 0 |
| Subscriptions from 1st July, 1891, to 1st July, 1892 ... ... ... ... ... ... | 19,409 | 4 | 4 |
| Proceeds of entertainments ... ... ... | 10,951 | 5 | 5 |
| From sale to Government of Nurses' Home at Quetta ... ... ... ... ... ... | 6,000 | 0 | 0 |
| From sale of furniture belonging to the same | 2,296 | 0 | 0 |
| Profits of nursing and ambulance classes held at Snowdon, Simla, by Surgeon-Captain Edwards ... ... ... ... ... ... ... | 1,181 | 8 | 3 |
| Refund from Dr. Davidson for expenses incurred on account of Sister Broadhead ... | 621 | 3 | 0 |
| Interest on investments ... .. ... ... | 8,143 | 7 | 0 |
| Total for the year ... ... ... ... | 59,516 | 0 | 0 |
| *Expenditure.* | | | |
| Expenses in maintaining homes and officers' hospitals, servants' wages, &c., from 1st July, 1891, to 1st July, 1892 .. ... ... | 4,359 | 1 | 0 |
| Travelling expenses of nurses in India and messing of two nurses on board troopship ... | 1,612 | 2 | 0 |
| Nurses' salaries from 1st July, 1891, to 1st July, 1892 ... ... ... .. ... | 10,567 | 8 | 0 |
| Reroofing with corrugated iron Murree Home and Officers' Hospital and out-houses ... | 3,973 | 10 | 0 |
| Building 5th ward to Officers' Hospital, Murree | 582 | 4 | 3 |
| New carpet and additional furniture to Nurses' Home and Officers' Hospital, Kasauli ... | 321 | 4 | 0 |
| Annual repairs ... ... ... ... ... ... | 217 | 3 | 8 |
| Advertising at half rates... ... ... ... | 40 | 5 | 0 |
| Invested* ... ... ... ... ... ... ... | 30,000 | 0 | 0 |
| Balance in hand on 1st July, 1892 ... ... | 7,843 | 2 | 7 |
| Total expenditure for the year ... ... | 59,516 | 0 | 0 |

* Making a total of Rs. 145,000 invested.

Simla, 11th July, 1892. Nora Roberts.

The receipts from patients, &c., formed but a comparatively small contribution to the working expenses, which, however, have been satisfactorily covered by the proceeds of entertainments and from other sources. The Homes are a great convenience for officers and their wives, who have mountain air, careful nursing, and medical attendance. The one at Kasauli is perched on a hillside, like a Swiss chalet, with a view down the valley and across to the pine woods of Upper Toba and the snowclad hills of Cashmere.

Whilst in the Homes the officers pay according to rank, each subaltern 5 rupees per diem; all above that rank 6 rupees per diem.

This is not more than a subaltern would pay at an hotel, and, as the prospectus of the Homes states, "These charges include servants, lights, fuel, medicines (which are supplied from the Government dispensary), nurses' services, and medical attendance. The only extras are stimulants, stationery, washing, ice, and any medicines not in the dispensary, and having to be procured from the chemist.

Charges for Nurses' Home: Each nursing sister, Rs. 2 per diem; each acting superintendent, Rs. 2 4a. per diem; each superintendent, Rs. 3. Extras as enumerated above.

A new regulation has come into force regarding the nursing sisters under Government; they can now cancel their five years' engagement by immediate payment of £30, the stated value of passage money on a troopship, or by giving six months' notice. This applies to sisters appointed in England; those taken on in India must give six months' notice or refund £15, outfit money. After finishing their five years' term of service, they have to sign an agreement if they wish to rejoin after their year's furlough (during which they draw two-thirds pay); but should they alter their minds about returning, they must refund all payments made to them during their "leave."

This is Lady Roberts's last year in India, and it would be gratifying if before next spring she could see the fund placed beyond reach of any future financial troubles.

### An Amusing Lecture.

By a Nurse.

A lecture delivered to nurses by a doctor on the subject of cholera is generally looked forward to as a serious, and even a possibly dull kind of affair. We should not, certainly, expect amusement but, rather instruction, delivered with dignity and received with humility. Dr. Heron's lectures prove that at least one man in London evolves comic positions from tragic elements. He is kindly devoting a great deal of time to put the finishing touches to the education of trained nurses. After informing them that outbreaks of cholera are associated with the presence of a certain little microbe, he has recommended the use of 5 per cent. carbolic acid (more familiar to old nurses as 1-20), and although he acknowledges the existence and merits of other disinfectants, this "faithful friend" ranks first in his confidence. As regards the origin and treatment of the disease, the lectures have taught us considerably less than the medical papers have admirably explained during many weeks past; in fact, even the daily journals have taught, directly and indirectly, various valuable details which Dr. Heron did not touch on.

Dr. Heron also omitted to tell us any of the safe things which could be rightly taken up by the nurse anxious to serve her patient whilst awaiting the doctor. The provision for alleviating sufferings by heat and rest, had but scant attention. Moreover we, as nurses, were startled by a warning not to prescribe for the patient, as in England the medical man was always within reach. All our probationers from their earliest days are imbued with this doctrine, therefore it can hardly be considered original; but still an accepted truth is inoffensive at any rate. Our instructor, with an inconsistency which is commonly considered characteristic of the other sex, went on to say that any nurse would be justified in giving twenty minims of laudanum, to be followed in half an hour by a second dose of fifteen, when an obstinate case of diarrhœa occurred. Of course, the "cholera scare" would be the justification, but not in our eyes a sufficient one for such a proceeding on the part of any person who had not a doctor's direct sanction for giving such doses of a poison, however high its reputation, to a patient for whom it was not personally ordered.

We find numbers of the general public prescribing for their friends to an alarming extent, but we should indeed be sorry if our nurses acted so unwisely.

The waterproof costumes designed for cholera nurses sound as unhygienic as they would be comfortless. They are to be, Dr. Heron says, of a material which the nurse can sponge over freely and frequently with the carbolic solution. They are to be short, of which we cordially approve, but the arms bare nearly from the shoulder don't sound nice. The nurse is to sponge herself with carbolic or perchloride (or both?), and she is to leave off shoes and stockings, and wear high boots of mackintosh. Feet are often trials to us, but any foregone experiences sink into insignificance before this picture of the cholera nurse's proposed woes! In fact, the summary of her personal care and ablutions leads to a probability that if they are literally carried out she will have little time to spare for those of her patient. But why is all this costume and elaborate personal care needful for cholera nursing? Can we not trust trained nurses who have learnt to take proper care of themselves whilst tending typhoid cases and leading them back to health and safety, to take equally faithful precautions in another disease which is infectious only in the same ways and from the same causes, neither more nor less?

Dr. Heron's description of his ideal typhoid or cholera bed is very unique. He wishes a sheet laid over a large mackintosh, which is to hang down on either side into a trough running round the bed. This trough is to contain carbolic and is to receive the discharges from the patient! Can this be a serious suggestion? What happens during the passage from bed to receptacle; where are the poisonous germs? And what about the patient's back and skin generally? Is re-absorption a fallacy, and what becomes of the first principle of trained nursing, viz., the sufferer's comfort? In cases of typhoid fever the preservation of the patient's skin is a source of anxiety from the beginning to the end of his illness. How is it to be kept sound if we exchange a smooth, dry, carefully placed sheet for a loose and therefore wrinkled one laid over an equally freely-arranged mackintosh, for, of course, no common-place "tucking in" of the bed-clothes would be feasible with a trough.

As regards the proposed enrolment of nurses who are willing to take charge of cholera, of course it can only apply to "Free Lances" in the profession, as hospitals and infirmaries, as well as other institutions, will have their own rules for their nurses. But these volunteers are kindly permitted to continue their usual avocations; in other words, to go on taking private cases, *but* they are to "hold themselves in readiness when called upon." Lucky private patient who innocently secures an attendant, to find later on that she belongs to the band of thoughtless enthusiasts who propose to do two things at once. In other words, to fly to a cholera patient at a moment's notice, and yet undertake the nursing of a person equally ill, though of a better-known disease, who will assuredly be unnerved and upset when he learns in some moment of confidence that his "own nurse" has pledged herself to break faith with him, if occasion should arise.

### Death in Our Ranks.

Nurse Agnes Howie died in a private ward at Ayr County Hospital, on September 3rd. Nurse Howie had worked for the last three years at Glasgow, on the private staff of the Sick Poor and Private Nursing Association, where she had endeared herself to Superintendent, nurses, and patients, by her kind and unselfish disposition. She was buried in Ayr Cemetery, and the Superintendents from Glasgow and Ayr, with some of her fellow nurses, were amongst the mourners.

Madame Miederlander died in Paris on the 6th inst. of cholera, contracted in the discharge of her duties in the hospital wards. Madame Miederlander was the wife of a printer, and was one of the first volunteers who came forward when the special wards for cholera patients were thought requisite for Paris.

### Royal National Pension Fund for Nurses.

The Council have received, as a free gift to the Junius S. Morgan Benevolent Fund, for the sole use and benefit of the poorer nurses, the sum of £200, with a request that it should be acknowledged as having been given in connection with Lord Tennyson's eighty-third birthday, by Sister Emma Durham, being the whole of the money received by her for services rendered as a hospital-trained nurse to the Poet Laureate.

### Appointment.

Clapham Maternity Hospital.—Miss Clara Hind has been appointed to the Matronship of the Clapham Maternity Hospital, in place of Miss Jean Millar, who has resigned. Miss Hind was trained at Nottingham General Hospital for two and a quarter years, and was afterwards Sister at Devonshire Hospital, Buxton. She enters on her new duties on September 17th.

### Everybody's Opinion.

*[Correspondence on all subjects is invited, but we cannot in any way be responsible for the opinions expressed by our correspondents. No communications can be entertained if the name and address of the correspondent is not given, or unless one side of the paper only be written on.]*

### GENERAL HOSPITAL, BIRMINGHAM.

We have received a letter from another "Sister" at this hospital, in which great praise is given to the improvements in nursing during the past seven years "which were wrought amid difficulties now non-existent, by a thoughtful kindness even they could not extinguish, but only incited to sterner toil in the noble undertaking." But this lady goes on to defend the practice of letting day and night nurses occupy adjoining bed-rooms, on the ground that "we should not expect from ladies sufficient noise to cause any inconvenience, and recreation hours should also certainly be spent outside the hospital walls." No doubt they should, but can this be possible in a town where rain and bad weather generally are not quite unknown? Must the day-probationer spend all her off-duty time out of doors to secure quiet to her companions who are on night duty? As regards noise too, this is relative, and we venture to say that no one makes a worse nurse for indulging in laughter and conversation when she has left the serious work of the wards for a short interval of relaxation. Most people have experienced the difficulty of keeping sufficient silence in a house to enable one person to sleep without disturbance during the daytime. Public opinion must decide which of our correspondents best understands the subjects of rest for those who need it, and recreation for those who have earned it!

### MILK AND SCARLET FEVER.

A correspondent says: "I am glad to read your interesting article on this subject. I wonder if other nurses think, as I do, that the insufficient and unclean water used for cleansing (?) milk cans is a great and unsuspected source of danger. At big dairies immense trouble and extreme heat are employed in the purifying of all vessels used to hold milk. But the small dealers in poor neighbourhoods have their cans washed in a very casual manner, generally by an irresponsible boy, on the pavement, and they are generally reversed for draining purposes and left standing on the said dirty pavement. Again, the small tin pail used for measuring the milk at the poor folks' doors is frequently set down on that dirty stone step whilst payment is made, and, of course, it is immersed in the milk for the next customer's supply."

### HOLIDAY HOMES FOR NORTHERN NURSES.—SCARBOROUGH.

A friend writes: "In answer to the enquirer in The Hospital for a Home for Nurses at Scarborough, I am glad to be able to tell her of one wherein I am now staying. It is presided over by two gentlewomen, who are Hospital Sisters, and who are most kind, and make us thoroughly at home and comfortable. I cannot speak too highly of it as a most refined home. Any ladies requiring rest, change, or nursing are received, and full particulars may be obtained by addressing the Lady Superintendent, 1, West Park Terrace, Scarborough."

### Presentation.

At a meeting of the weekly board of the Royal Free Hospital, held on Thursday last, the Chairman, Mr. Edward Ford North, presented Mr. Conrad W. Thies, the secretary, who will shortly be married to Miss E. Storey, with a handsome polished oak case of knives, forks, and spoons, which had been subscribed for by the members of the Committee of Management as a mark of their appreciation of the valuable services rendered by him to the institution, and as an expression of their best wishes for his future happiness. Mr. Thies has also been the recipient of wedding gifts from members of the medical and nursing staff and the officers of the hospital; and in leaving for his well-earned holiday he will carry with him the heartiest good wishes of all in the institution which he serves with so much energy and zeal.

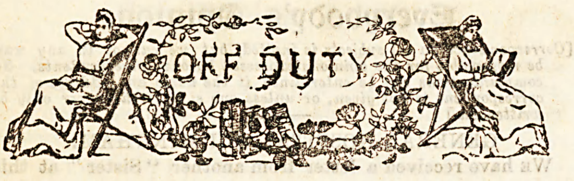

### The Result of a Chill.

(*Continued from page* clxxiv.)

Since the artist's sojourn at Hollacombe six water-colour sketches and two oil paintings had been despatched to Mr. Blatch, the dealer. Three of the sketches were by Hilda, and the remainder were Sybil's. They were sent with large hopes of acceptation, for they represented many weeks of assiduous toil, and were the highest examples of the girl's budding powers. Each subject was judiciously selected, painted direct from nature, and carefully worked up in all detail.

"Oh, if only they all sell!" said Hilda, when a friendly farmer drove away to Moreton-Hampstead with the pictures in his cart. "How much will they make, Sybil?"

"As a modest computation, dear, I honestly think they are worth thirty pounds. If they bring us twenty, let us be satisfied. They are first productions, remember; still, I don't think them absolutely amateurish."

"I think your atmosphere is simply lovely," remarked Hilda. "How much you have improved since you came here. There is nothing like dogged determination, is there? You said you would get that filmy evening mist in the Kestor picture or perish, and you caught it admirably."

"Will Mr. Blatch think so?" asked Sybil, with a doubtful curl of the upper lip.

"I hope so. I suppose we sha'n't hear for a week. Let us make tea."

They seated themselves in the dusk of autumn to a meal of toast, eggs, Devonshire cream, and tea, laid on a cheap deal table, transmuted by means of enamel paint and an oriental table-cloth to a less unsightly piece of furniture. While they sat in wholesome enjoyment of their country fare, one side of the deep embrasure of the window reflected the lurid gleam of the sun sinking in a stormy mist of rain that scudded before a freshening gale from the west. It was the beginning of one of those hurricanes that sweep Dartmoor with fierce yells, and make awful elemental conflict beneath rolling clouds of deepest purple. At such times the mighty waste is a place of sublime and weird grandeur. The jagged peaks of tors are obscured by inky palls, with the grey streaks of the terrific downpour in the foreground, and breaks of an angered, steely firmament between drenched hill-sides. Shrieking as it drove the rain in slanting veils from the summits to the cleaves, the wind caught Hollacombe in its frantic rush, and made the sturdy old walls quiver. The brown beech leaves before the house parted from the groaning boughs in a whirl, and raced by the windows; raindrops fell with a hiss on the fire of peat and logs. The slope of the hill was blotted out by the flying deluge, and the ponies came down from the heights to huddle in a group in the little lea before the coppice of dwarf oaks.

Aroused from their table by the sudden tumult of wind and rain, the girls stood by the window to gaze at this scene of wild animation. While they watched, the bent figure of a man battling with the gale passed like a phantom before the window. Then came a vigorous knocking with a stick on the oak door, and the sound of a cloak being shaken.

"A lost tourist," said Sybil. "The hump on his back was a knapsack. What a night to be out."

She went to the door, and drew the bolts. A dreadfully sodden and mudstained mortal was wringing the water out of a cloth cap.

"Pardon me for troubling you," he began, looking surprised at the unexpected apparition of a young lady. "I'm on a walking tour, and I have been trying to find the track to Chagford since the storm began. I have lost sight of all the landmarks in this downpour, and the map is no use to me."

He produced a soaked ordnance sheet, and unfolded it against the wall. "Here is my point," he said; "how am I to strike it from here?"

"I am afraid," replied Sybil, "you will have great trouble in doing so. I can direct you, but it would be very difficult to find your way in the dark, and it is over five miles from here. I am afraid you are soaked," she added, glancing at his streaming cheeks and dripping beard.

"I don't think a man could be much wetter," he answered, as he dabbed his face with a damp pocket handkerchief. "Five miles, you say? I shall be washed away before I can get to an hotel. I suppose it is absurd to ask if a bed can be had nearer than Chagford?"

"There is not another house for two miles, and that is a very dilapidated cottage, inhabited by a half-demented old woman. But wait a moment, please?"

She went to the sitting-room and said to Hilda: "It's a gentleman, dear, drenched through. He wants to find Chagford. It's impossible on such a night."

"Utterly," returned Hilda. "Ought we to ask him to shelter here?"

Sybil looked perplexed.

"We must ask him in common decency," she said. "We can't turn him away in this frightful deluge. He is unmistakably a gentleman."

She hesitated for some reply from Hilda, who, while she recognised the duty of entertaining strangers, was not sure whether the duty devolved upon unprotected spinsters.

"We cannot keep him waiting," murmured Sybil, returning to the door. "Will you come in?" she said. "We shall be glad to give you a room for the night. You can't think of going further in this storm."

"I am sure your are extremely kind," remarked the tourist. "I am afraid I shall be putting you to inconvenience."

"Not at all. Please come in."

(*To be continued.*)

### Notes and Queries.

To Correspondents.—1. Questions or answers may be written on post-cards. 2. Advertisements in disguise are inadmissible. 3. In answering a query please quote the number. 4. A private answer can only be sent in urgent cases, and then a stamped addressed envelope must be enclosed. 5. Every communication must be accompanied by the writer's full name and address, not necessarily for publication. 6. Correspondents are requested to help their fellow nurses by answering such queries as they can.

### Answers.

*Guild of St. Barnabas* (Country Nurse).—Address the Secretary, Miss Wood, Percy Street, Tottenham Court Road.

*Probationers* (M. E. D.).—You are not eligible for any hospital until you are older. The age is 25 for adult and 21 for children's hospitals. Try provincial or fever hospitals.

*Nurse Shipman* (Paralysis).—You had better consult the medical attendant, as the subject you write about is certainly most unsuitable for discussion in a newspaper. An experienced nurse could give you practical hints, but, under the circumstances, you should ask the doctor's advice.

*W. A. H.* (Male Nurses).—We hope to print a letter next week which will give the information you desire.

*Pro. Dorothy* (Certificate).—Write to Matron, London Hospital, Whitechapel, for regulations. Three years' general training is required to make a nurse eligible for a naval or military hospital.

*Dispensing* (Spes.)—Get "Pharmaceutical Journal," published 17, Bloomsbury Square.

### Wants and Workers.

Nurse Fry, who is starting district nursing amongst the poor in their own homes, at Weston, would be very grateful for a large-sized water-pillar (in good condition). She would also gladly receive and acknowledge anything else in the way of invalid comforts and appliances, as she is anxious to get things to carry on the work efficiently through the winter.—Address *Nurse Fry, The Hospital, Weston-super-Mare.*

